# Concepts and issues related to adolescent health in nursing education*

**DOI:** 10.1590/1518-8345.6166.3651

**Published:** 2022-09-23

**Authors:** Silvia Helena De Bortoli Cassiani, Bruna Moreno Dias, Martha Patricia Bejarano Beltran, Lucy Marcela Vesga Gualdrón, Taycia Ramírez Pérez, Germania Marivel Vargas Aguilar, Rudi Amalia Loli Ponce, Angela Rocio Cornejo Valdivia

**Affiliations:** 1 Organización Panamericana de la Salud, Departamento de Sistemas y Servicios de Salud, Washington, DC, Estados Unidos da América.; 2 Universidad Nacional de Colombia, Facultad de Enfermería, Bogotá, DC, Colômbia.; 3 Universidad de Guayaquil, Escuela de Enfermería, Guayaquil, Provincia del Guayas, Equador.; 4 Universidad Nacional Mayor de San Marcos, Escuela de Profesional de Enfermería, Lima, Provincia de Lima, Peru.

**Keywords:** Adolescent Health, Nursing, Curriculum, Education, Nursing, Diploma Programs, Teaching, Health Human Resource Training

## Abstract

**Objective:**

analyze the structure of teaching programs and contents of the adolescent health and its development in nursing education.

**Method:**

descriptive observational study conducted in Colombia, Ecuador and Peru. Data was collected by the Instrument for (self-)evaluation of the adolescent health and development component in undergraduate nursing programs, and investigated using descriptive statistics.

**Results:**

A total of 95 nursing schools participated in the study (84.1% of the total). Of the faculty responsible for teaching contents relating to adolescent health, 31.6% had no specific training on the topic and 18.9% had no educational/pedagogical training. Behavior, gender identity and sexual orientation, bullying and cyberbullying, use of digital technologies, partner violence, parenting, and pubertal delay were the main topics which could be incorporated into nursing education.

**Conclusion:**

the faculty of the schools analyzed have limited education in pedagogical/educational topics and specific adolescent health issues. Curricula should be updated, including knowledge of laws and policies, thus expanding nurses’ ability to generate, analyze and use data for decision making.

## Highlights

(1) 31.6% of faculty have no specific education in adolescent health.

(2) 18.9% of faculty have no educational/pedagogical training.

(3) Progress is needed in the use of active methodologies and interactive multimedia.

(4) Knowledge of laws and policies for the adolescent population must be expanded.

(5) Current and relevant adolescent health issues need to be addressed in nursing education.

## Introduction

Adolescence is an important stage of human development, as adolescents face diverse factors such as physical growth, hormonal changes, sexual development, new emotions, expansion of cognitive skills, and moral and relationship development^1^. Thus, this developmental stage requires specific communication approaches and caregiving skills^2^.

Adolescents represent 16% (1.2 billion) of the world’s population and are an important group for economic and social development. Although considered a healthy group, globally adolescents face barriers in accessing health services. An estimated 0.9 million adolescents died in 2019 from preventable or treatable conditions, thus investing in the health of this population is essential to ensure the health of future generations and advance universal health^1^.

Latin America and the Caribbean region lack investment, and sustainability of interventions aimed at adolescents, including actions to ensure that health systems and services respond to their needs^3^. Thus, services are expected to act in an intersectoral manner to constitute a welcoming space for this population; approaching them and effectively adopting actions aimed at their particular needs^4^.

Identifying and addressing gaps in care, such as access barriers in health services related to age, poverty, geographic location, disability, ethnicity, conflict, sexual orientation, gender identity and religion are fundamental in sexual, reproductive, maternal, neonatal, newborn and adolescent health^5^. In this regard, the Plan of Action for the Health of Women, Children and Adolescents 2018-2030, of the Pan American Health Organization (PAHO), suggests actions along four strategic lines: strengthening a transformative policy environment to reduce inequalities; promoting universal, effective and equitable health and wellness; expanding equitable access to comprehensive, integrated and skilled health services, centered on the person, family and community; and strengthening information systems for the collection, availability, accessibility, quality and dissemination of strategic information^6^.

At the professional level, one must invest in education and training; planning, management and regulation of health professionals and working conditions; leadership and governance; and service provision^5^. Given the number and role of nurses in health services and systems, strengthening the nursing workforce requires policy priorities that focus on investing in education, employment, leadership, and maximizing their contributions in work settings, as advocated in the Global Strategic Directions on Nursing and Midwifery 2021-2025^7^.

The Region of the Americas have significant disparities in nursing education, expressed in the discrepancies in the proportions of professionals in certain geographic regions or types of services and also in the levels of training and competencies of these professionals, factors that affect the capacity of the nursing workforce and the quality of care provided^8^. Given this context, improving nursing education will have a positive impact on population health.

Nursing education programs also must be capable of ensuring effective student learning, as well as meeting quality standards and health care needs^7^.

Attention to curriculum is paramount in the context of the recent pandemic, which has imposed significant changes in the teaching model and impacted the quality of education^9^. If, on the one hand, the COVID-19 pandemic imposed important changes in education^9^, on the other, it points out the need for health and education systems to turn their attention to vulnerable groups, making it imperative for nursing education to become a space to respond to health inequalities^10^.

To address these inequalities, health professionals and services must develop competencies and skills as not to become an obstacle to advancing universal health for adolescents^2^. Nurses and other profesionals, also via continuing education strategies, should develop specific competencies focused on adolescent health promotion^11^, especially for populations in situations of vulnerability.

Given the need for actions and strategies aimed at improving adolescent health, this paper examines how nursing education has been developed and what training and qualification nurses’ needs are in terms of adolescent health. To do so, it analyzes the structure of nursing education programs and the contents of the adolescent health and its development in undergraduate nurse training.

## Method

### Study design, place and period

This is a cross-sectional and observational study conducted in Colombia, Ecuador and Peru, in 2021.

### Participants

All nursing schools in the three countries were considered eligible, with a total of 113 schools, of which 47 in Colombia, 24 in Ecuador and 42 in Peru^12^. Convenience sample resulted in 95 schools in the three countries, corresponding to a participation rate of 84.1%. Participation by country was: Colombia 39 (82.9%), Ecuador 23 (95.8%) and Peru 33 (78.6%).

### Instruments

Tool to assess the adolescent health and development component in the pre-service education of health-care providers, developed and validated by the World Health Organization (WHO) in Spanish, was applied. The questions were structured in four sections: General information about the institution and the nature of the curriculum/course; Information on courses or a curriculum track dedicated to adolescent health; Review of the foundation of adolescent health care; and Review of topics linked to management of specific clinical situations in adolescents^2^. Survey responses were voluntary. Besides the instrument questions, participants could submit a link and/or a document of their School of nursing curriculum.

### Data collection

Invitation was sent by email to all schools registered in the PAHO Directory of Nursing Schools to participate in the study^12^. Prior to data collection, the authors held meetings with the participants to present the project and clarify any doubts. All 113 schools registered in the Directory were invited to participate in the orientation meetings, however attendance was optional. As an additional resource, the authors provided a video presentation of the project and survey response guidelines.

The dean, course coordinator and faculty of disciplines in related areas of adolescent’s health were invited to respond the instrument.

Invitation was sent to the schools, regardless of their participation in the orientation meetings. Response of the instrument was requested in within one week, with a reminder sent after this deadline and a new reminder after 15 days. Moreover, contacts were established with collaborating universities to further disseminate the survey.

Data was collected by the researchers between October and November 2021, using the SurveyMonkey tool. Average time to complete the instrument was one and a half hours.

### Data processing and analysis

The answers obtained were entered into a spreadsheet, forming an electronic database, and then exported export to IBM SPSS Statistics software, version 28. The nursing programs were characterized by descriptive analysis. All 95 schools were considered in the analysis, even though some items were not answered by all participants.

### Ethical aspects

The project was approved by the Pan American Health Organization’s Ethics Review Committee under protocol no. 0435.01.

All participants had access to the informed consent form on the survey participation page, before their approval to participate in the study. Participants who selected the “I do not agree” option had their participation suspended.

## Results

A total of 95 nursing schools from three countries participated in the study: 39 from Colombia (41.1%), 23 from Ecuador (24.2%) and 33 from Peru (34.7%). Five-year programs were the most frequent (46.3%), followed by four-and-a-half-year programs (31.6%) and four-year programs (20%).

Schools of nursing are concentrated in the capital and major cities, pointed out the disparity in the distribution of schools and training opportunities in all regions of the countries.

Curricula are formally approved by national authorities in 98.9% of the courses. The content of the courses is determined by both the working groups (96.8%) and the coordinator. Curriculum is updated periodically, without repeating from one year to the next (86.3%), considering local regulations, laws and the health context (95.8%). Of the 95 schools, 96.8% reported faculty accreditation and performance evaluation processes, whereas 98.9% claimed student participation.

Regarding the curriculum, in 92.6% of the schools the competencies are predefined and formalized. Importantly, 17.9% of the courses lack teaching methods adapted to the objectives, 26.3% of the faculty lack educational and pedagogical training, and 21.1% of the faculty do not receive orientation for competency-based education.

Clinical practices (85.3%), case studies (81.1%) and use of simulated patients (61.1%) are the most frequent didatic methods used. Simulations (28.4%) and interactive multimedia (53.7%) are less frequent.

Evaluation is often conducted by structured practical exams (98.9%), written exams (97.9%) and direct observation. In 14.7% of the schools, patient history evaluation is not used as an evaluation method.

Most schools (n=86) claimed to have a course or module dedicated to adolescent health, although 45.3% of them offer it as part of other disciplines, such as: children’s health (83.2%), community/family nursing (77.9%) and women’s health (74.7%) (Table 1).

**Table 1:** Adolescent health teaching, and whether there is a subject or module dedicated to adolescent health in the content of selected nursing schools (n=95). Colombia, Ecuador and Peru, 2021

	Yes	No	Do not know
	n(%)	n(%)	n(%)
Adolescent health and development are taught “independently.”	42 (44.2)	43 (45.3)	1 (1.1)
Adolescent health and development is part of other disciplines:			
• Women’s health	71 (74.7)	15 (15.8)	0 (0)
• Children’s health	79 (83.2)	6 (6.3)	1 (1.1)
• Community/family nursing	74 (77.9)	11 (11.6)	1 (1.1)
• Mental health nursing	68 (71.6)	17 (17.9)	1 (1.1)
A combination of the above.	66 (69.5)	18 (18.9)	2 (2.1)
There is a person or group in charge of coordinating all disciplines related to adolescent health and development.	62 (65.3)	24 (25.3)	0 (0)
Faculty in charge meet together to coordinate the content of their disciplines.	75 (78.9)	11 (11.6)	0 (0)
Faculty responsible for teaching adolescent health has received specific training on the subject.	56 (58.9)	30 (31.6)	0 (0)
Faculty responsible for teaching adolescent health has been trained in education/pedagogy.	66 (69.5)	18 (18.9)	2 (2.1)

Of the faculty responsible for adolescent health teaching, 31.6% had no specific training in the topic and 18.9% had no training in education/pedagogy.

Of the schools that reported not having a discipline or module dedicated to adolescent health (n=7), most reported interest in including adolescent health in their curricula and educational activities; however, they believe that their faculty need more training to improve their competence in this area. In the opinion of these schools, the topic should be part of other disciplines, such as: Women’s Health, Community/Family Nursing, Mental Health Nursing and Children’s Health.

Of the basic concepts related to adolescent health and development, those related to understanding adolescence; practice environment and communication skills; laws and policies affecting adolescent health care delivery are most often taught in schools (Table 2).

**Table 2:** Basic concepts related to adolescent health and development (n=95). Colombia, Ecuador and Peru, 2021

	Yes, this subject is taught in our curriculum	No, this subject is not taught, but it should be	This subject is not taught and is not relevant
	n(%)	n(%)	n(%)
**Understanding adolescence**			
Definitions and concepts of adolescence	90 (94.7)	2 (2.1)	0 (0)
Normal growth and puberty, including implications for body image	86 (90.5)	6 (6.3)	0 (0)
Cognitive development	83 (87.4)	9 (9.5)	0 (0)
Psychosocial development	86 (90.5)	6 (6.3)	0 (0)
Sexuality development	88 (92.6)	3 (3.2)	1 (1.1)
Assessment of developmental stages	89 (93.7)	3 (3.2)	0 (0)
Protective and risk factors related to adolescent development	81 (85.3)	11 (11.6)	0 (0)
Epidemiology of adolescent, health outcomes and health-related behavior	72 (75.8)	20 (21.1)	0 (0)
Local attitudes, beliefs, and practices regarding adolescents	68 (71.6)	24 (25.3)	0 (0)
**Consultation environment and communication skills**			
How to create an atmosphere of trust during consultation (privacy, confidentiality)	67 (70.5)	25 (26.3)	0 (0)
Conduct an anamnesis, including a psychosocial evaluation	77 (81.1)	14 (14.7)	1 (1.1)
Physical examination	87 (91.6)	5 (5.3)	0 (0)
Factors influencing effective communication with adolescents	68 (71.6)	24 (25.3)	0 (0)
Communication with parents	70 (73.7)	21 (22.1)	1 (1.1)
Gender norms in adolescent health care	66 (69.5)	25 (26.3)	1 (1.1)
Health education	85 (89.5)	7 (7.4)	0 (0)
Motivational assessment	56 (58.9)	35 (36.8)	1 (1.1)
**Laws and policies affecting adolescent health care provision**			
National laws and policies affecting adolescent health care provision.	67 (70.5)	25 (26.3)	0 (0)
Health care focus based on human rights	77 (81.1)	15 (15.8)	0 (0)
Ethics	81 (85.3)	11 (11.6)	0 (0)
Assessment of adolescent decision-making competence	60 (63.2)	31 (32.6)	1 (1.1)
School health and the role of schools in health promotion	75 (78.9)	17 (17.9)	0 (0)
Adolescent health promotion in the community	83 (87.4)	9 (9.5)	0 (0)

As for the topics that are not taught, but should be, the most frequently cited were: motivational interviewing (36.8%); assessment of the adolescent’s competence to make decisions (32.6%); how to create an atmosphere of trust during consultation (26.3%); gender norms in adolescent health care (26.3%), national laws and policies affecting adolescent health care service provision (26.3%); local attitudes, beliefs and practices regarding adolescents (25.3%); factors influencing effective communication with adolescent users (25.3%); communication with parents (22.1%); epidemiology of health outcomes and adolescent health-related behavior (21.1%); and school health and the role of schools in health promotion (17.9%).

Related to the management of adolescent-specific clinical situations (Table 3), the most frequent topics focused on adolescent needs taught in more than 50% of the schools are: immunization; anamnesis of sexual and reproductive health; normal menstruation and menstrual hygiene; sexual attitudes and behaviors; body image issues and eating disorders; prevention of sexually transmitted infections (STI) including human immunodeficiency virus (HIV); adolescent pregnancy, prenatal and postnatal care; menstrual pain; menorrhagia/metrorrhagia, irregular menstruation; nutrition and healthy eating, nutritional needs; precocious puberty; STI diagnosis including HIV; overweight and obesity; developmental disorders; tobacco use; alcohol use and related disorders; anemia; drug use and related disorders; self-harm and suicide; and skin conditions.

**Table 3:** Content related to the management of adolescent-specific clinical situations (n=95). Colombia, Ecuador and Peru, 2021

	Yes, the subject is included, and focused on the adolescent	Yes, the subject is included, but not focused on the adolescent	No, the subject is not included, although it should be, focusing on the adolescent	No, the subject is not included and is not relevant
	n(%)	n(%)	n(%)	n(%)
Male pubertal delay	46 (48.4)	17 (17.9)	22 (23.2)	4 (4.2)
Female pubertal delay	47 (49.5)	16 (16.8)	22 (23.2)	4 (4.2)
Short stature	46 (48.4)	21 (22.1)	20 (21.1)	2 (2.1)
Precocious puberty	52 (54.7)	13 (13.7)	21 (22.1)	3 (3.2)
Immunization	66 (69.5)	20 (21.1)	2 (2.1)	1 (1.1)
Abdominal pain	43 (45.3)	32 (33.7)	11 (11.6)	3 (3.2)
Anemia	49 (51.6)	32 (33.7)	5 (5.3)	3 (3.2)
Fatigue	39 (41.1)	36 (37.9)	10 (10.5)	4 (4.2)
Headache	41 (43.2)	35 (36.8)	9 (9.5)	4 (4.2)
Skin conditions	48 (50.5)	14 (14.7)	20 (21.1)	7 (7.4)
Poor vision	36 (37.9)	35 (36.8)	13 (13.7)	5 (5.3)
Poor hearing	36 (37.9)	35 (36.8)	14 (14.7)	4 (4.2)
Respiratory infections, pneumonia, asthma	45 (47.4)	35 (36.8)	5 (5.3)	4 (4.2)
Orthopedic problems	30 (31.6)	39 (41.1)	16 (16.8)	4 (4.2)
Endemic diseases	31 (32.6)	41 (43.2)	13 (13.7)	4 (4.2)
Dental problems and oral health	35 (36.8)	32 (33.7)	16 (16.8)	6 (6.3)
Gender identity and sexual orientation	43 (45.3)	20 (21.1)	20 (21.1)	6 (6.3)
Sexual attitudes and behaviors	57 (60)	15 (15.8)	11 (11.6)	6 (6.3)
Anamnesis of sexual and reproductive health	61 (64.2)	17 (17.9)	9 (9.5)	2 (2.1)
Normal menstruation and menstrual hygiene	58 (61.1)	18 (18.9)	11 (11.6)	2 (2.1)
Menstrual pain	55 (57.9)	19 (20)	13 (13.7)	2 (2.1)
Menorrhagia/metrorrhagia, irregular menstruation	54 (56.8)	23 (24.2)	9 (9.5)	3 (3.2)
Diagnosis of STI* including HIV^†^	52 (54.7)	27 (28.4)	7 (7.4)	3 (3.2)
Prevention of STI* including HIV^†^	55 (57.9)	24 (25.3)	7 (7.4)	3 (3.2)
STI* treatment	45 (47.4)	31 (32.6)	10 (10.5)	3 (3.2)
HIV^†^ treatment	45 (47.4)	33 (34.7)	8 (8.4)	3 (3.2)
Special problems of adolescents infected with HIV^†^ in the perinatal period	36 (37.9)	34 (35.8)	14 (14.7)	5 (5.3)
Preputial problems	28 (29.5)	29 (30.5)	25 (26.3)	7 (7.4)
Acute scrotal pain	24 (25.3)	28 (29.5)	29 (30.5)	8 (8.4)
Contraception, including emergency contraception	41 (43.2)	30 (31.6)	13 (13.7)	5 (5.3)
Teenage pregnancy, prenatal and postnatal care	55 (57.9)	28 (29.5)	3 (3.2)	3 (3.2)
Adolescent parenthood	40 (42.1)	22 (23.2)	23 (24.2)	4 (4.2)
Safe abortion	34 (35.8)	34 (35.8)	15 (15.8)	6 (6.3)
Preventive interventions for safe sexual practices	46 (48.4)	23 (24.2)	18 (18.9)	2 (2.1)
Epidemiology of adolescent chronic diseases	43 (45.3)	23 (24.2)	19 (20)	4 (4.2)
Psychosocial problems and chronic conditions	44 (46.3)	25 (26.3)	16 (16.8)	4 (4.2)
Adherence to treatment	33 (34.7)	32 (33.7)	18 (18.9)	6 (6.3)
Transition to adult care	32 (33.7)	19 (20)	29 (30.5)	9 (9.5)
Assessment of mental health problems	43 (45.3)	33 (34.7)	10 (10.5)	3 (3.2)
Depression	45 (47.4)	34 (35.8)	8 (8.4)	2 (2.1)
Body image issues and eating disorders	56 (58.9)	24 (25.3)	8 (8.4)	1 (1.1)
Self-harm and suicide	48 (50.5)	30 (31.6)	9 (9.5)	2 (2.1)
Anxiety disorders and phobias	42 (44.2)	35 (36.8)	9 (9.5)	3 (3.2)
Attention Deficit Hyperactivity Disorder (ADHD)	33 (34.7)	35 (36.8)	16 (16.8)	5 (5.3)
Mental disorders and delirium	29 (30.5)	39 (41.1)	15 (15.8)	6 (6.3)
Developmental disorders	50 (52.6)	25 (26.3)	10 (10.5)	4 (4.2)
Use and misuse of digital technologies	31 (32.6)	24 (25.3)	28 (29.5)	6 (6.3)
Epidemiology of substance use	46 (48.4)	25 (26.3)	13 (13.7)	5 (5.3)
Tobacco use	50 (52.6)	25 (26.3)	11 (11.6)	3 (3.2)
Alcohol use and related disorders	49 (51.6)	26 (27.4)	11 (11.6)	3 (3.2)
Drug use and related disorders	48 (50.5)	28 (29.5)	9 (9.5)	4 (4.2)
Medication and self-medication of mental disorders	28 (29.5)	39 (41.1)	17 (17.9)	5 (5.3)
Nutrition and healthy eating, nutritional needs	53 (55.8)	31 (32.6)	3 (3.2)	2 (2.1)
Overweight and obesity	51 (53.7)	34 (35.8)	2 (2.1)	2 (2.1)
Underweight and starvation	28 (29.5)	36 (37.9)	19 (20)	6 (6.3)
Malnutrition and micronutrient deficiencies	40 (42.1)	39 (41.1)	7 (7.4)	3 (3.2)
Bullying and cyber bullying	22 (23.2)	26 (27.4)	33 (34.7)	8 (8.4)
Family and intimate partner violence	38 (40)	40 (42.1)	10 (10.5)	1 (1.1)
Youth and partner violence	37 (38.9)	22 (23.2)	24 (25.3)	6 (6.3)
Sexual assault	37 (38.9)	33 (34.7)	16 (16.8)	3 (3.2)
Injuries caused by traffic accidents	34 (35.8)	34 (35.8)	14 (14.7)	7 (7.4)
Other injuries	30 (31.6)	33 (34.7)	19 (20)	7 (7.4)
Physical activity and sports	42 (44.2)	36 (37.9)	7 (7.4)	4 (4.2)

*Sexually Transmitted Infections (STI); ^†^Human Immunodeficiency Virus (HIV)

Among those topics that should be included, the most frequently cited are: cyberbullying and bullying (34.7); transition to adult care (30.5); acute scrotal pain (30.5); use and misuse of digital technologies (29.5); preputial problems (26.3); youth and partner violence (25.3); adolescent parenthood (24.2); female pubertal delay (23.2); male pubertal delay (23.2); precocious puberty (22.1); skin conditions (21.1); gender identity and sexual orientation (21.1); and short stature (21.1).

## Discussion

In analyzing the offer and distribution of nursing courses, one must consider the need to expand education opportunities, which implies understanding the local demand for new professionals, in a scenario of significant differences in the nursing workforce composition, as in the relations between nurses and associate professional nurses, which is 19.36 in Colombia, 49.55 in Ecuador and 43.20 in Peru^13^.

Regarding the teaching program structure, curricula should be competency-based and ensure effective learning aligned with the population’s health needs^7^.

Curriculum preparation and updating, according to the schools, is a collective process, that involves various participants increasing the chances of a successful implementation^14^. The schools also reported systematized accreditation and evaluation processes, essential for ensuring program quality^15^, if criteria for using a rigorous method of program evaluation are met^16^.

Quality teaching requires permanent updating and training of the faculty, to ensure proper training in the best pedagogical methods and technologies, with proven clinical experience in the areas^7^.

In adolescent health, the use of evidence-based research in health education approaches is a challenge, thus demanding specific training^2^.

Besides better qualified faculty, nursing schools should incorporate different and appropriate teaching and evaluation methods to meet student learning needs^7^ ^,^ ^17^.

By adopting new and varied teaching methods, programs can benefit from creating a clinical learning environment based on small groups and interaction between students, educators and nurses^18^, based on active learning methodologies.

Activities including clinical practice and greater interaction between students and the practice setting, such as the community context, allow students to understand the role of the nurse, fostering professional and personal development^19^; therefore, such activities should be encouraged and improved.

To incorporate teaching methods, schools must also consider the student profile and include information and communication technologies^17^.

Activities that promote digital education, the use of interactive multimedia and simulation sessions have been more widely used in nursing education; however, more investment is needed to ensure faculty support and effective use of resources^7^.

Virtual simulation could facilitate theoretical and clinical development, with positive student feedback. It is even a useful resource in the context of the COVID-19 pandemic, providing learning experiences and increasing the student’s confidence in their skills, besides being a method to evaluate student performance, when the objectives of the activity are clearly defined^20^ ^-^ ^21^.

The poor incorporation of multimedia teaching methods reinforces the need for nursing to accelerate its digital transformation processes, both in teaching and in the contexts of research and practice^22^.

Although digital education for healthcare professionals can help them develop competencies, one must consider that its outcomes vary due to factors such as modality, method of instruction, evaluation methods, learning pedagogies, population and subject/discipline^23^.

The virtual nursing education imposed by the pandemic, has shown different experiences for first- and final-year students, as those at the beginning of the course predominantly learn theoretical concepts, whereas those finalizing the course have activities focused on clinical training. Besides these differences, the quality of student-professor interaction is an essential point of attention when incorporating such technologies^24^.

As for the teaching of adolescent health topics, most schools have a module dedicated to the subject, although not always independently, as some courses include it as part of other subjects, such as children’s health, community/family nursing and women’s health.

The teaching of topics related to adolescent health has been developed both: independently an in a integrated manner, as in medical courses. Independent teaching emphasizes the importance of the subject as an area of learning, but students may forget the content taught throughout the course. Integrated teaching with other subjects involves more repetition and reinforcement, approaching the subject as topics of interest and not only complementary^2^.

Although considered a healthy age group, adolescents are affected by a number of conditions that are sometimes ignored. Road traffic injuries, diarrheal diseases, tuberculosis, interpersonal violence and self-harm are the main causes of mortality; whereas their main health problems are behavioral, such as alcohol and tobacco use, unprotected sex, poor diet, inadequate physical activity, or conditions such as tuberculosis and mental disorders^1^. These conditions are usually addressed in nursing courses, although some to a lesser extent, such as traffic accidents and other injuries.

Importantly, topics related to the management of clinical situations appear in the curriculum, but are not adolescent-centered, which may prevent students from understanding the topic as relevant to this population.

The emphasis given to clinical conditions relevant to adolescent health is especially important considering the particularities of this group and their user profile. Demand for health services can be affected by several conditions, such as gender, education and social profile^25^, with community services being a key point of care, generally due to illness, to the detriment of health promotion actions^4^. Among the factors limiting this population’s access to health services are long waiting periods, lack of care prioritization, and geographic barriers^4^.

As for comprehensive adolescent care, besides issues related to health accessibility, one must consider the complexity of actions and the possibility of intersectoral interventions, as observed in alcohol consumption, in which the relationship between health service practices and school, and the harmonized family relationship are protective factors for adolescents^26^.

Among the neglect topics are bullying and cyberbullying. These are important issues for health professionals, due to its prevalence and potential health harms to adolescents, since cyberbullying has a prevalence of up to 35.4%, and victims present more emotional and psychosomatic problems, social difficulties, moderate to severe depressive symptoms, substance use and suicide ideation and attempts^27^. Other studies point out the increased risk of self-harm, suicidal behavior^28^ and depression in adolescent victims of cyberbullying, highlighting the need for prevention and management actions^29^.

Complementarily, the use and misuse of digital technologies occupies ranks second among content that should be taught. Excessive use of digital technologies is associated with decreased well-being, with adolescents being the most vulnerable group^30^. Smartphone addiction and overuse has been shown to be a concerning factor due to its effects on adolescent health, such as decreased hours of sleep, neck pain and mental disorders. Moreover, excessive use of social networks has been associated with decreased self-esteem and body satisfaction, elevated risk of cyberbullying, increased exposure to pornographic material and risky sexual behaviors^31^.

Youth and partner violence are issues yet to be addressed. In 2018, one in four adolescent girls aged 15-19 who were married or in a relationship experienced partner violence^1^.

Despite the high prevalence of adolescents who experience some type of violence (sexual, physical, psychological or institutional), the issue is often silenced and made invisible due to the reproduction of social gender norms^32^. It is worrying that this group has few support networks and do not consider health professionals as a support source^33^.

Combating violence against adolescents requires discussions on the content by different institutions and the implementation of intersectoral actions, including the strengthening of public health actions and the establishment of an effective link with health professionals, to create a support and protection network^34^ ^-^ ^35^, considering, among other things, the impact of the COVID-19 pandemic on the prevalence of violence^35^.

Another topic of interest is teenage parenthood. Adolescent parents disproportionately come from single-parent families and low parental socioeconomic status. As a result, children of adolescent parents are at increased risk for prematurity, low birth weight, and psychological disorders^36^ ^-^ ^37^.

From a clinical perspective, issues such as delayed or precocious puberty can also trigger emotional and psychosocial disorders; thus, timely treatment and support for adolescents and families are necessary^38^.

Gender identity and sexual orientation are also topics related to possible physchological distress. Adolescents victims of intolerance to gender identity and sexual orientation are at increased risk of lack of opportunities, dropping out, loss of family ties and suicidal behavior^39^. Young people have specific health needs and sometimes face barriers in accessing health services, health promotion and wellness actions^40^.

Given the problems outlined above, school nursing should be adopted to expand health knowledge and health-promoting behaviors is advocated^41^, expecially for vulnerable population with lower scores in health knowledge^42^.

School nurses can support this population by establishing an effective bond, listening carefully to their feelings and health needs, intervening early and continuously, in collaboration with the family, school and health services, based on comprehensive care^43^. Studies show that, from the perspective of the educational system, school nurses contribute to improve health, reduce absenteeism and improve student performance^44^. Hence, WHO supports the creation of school-based health centers as an important community resource to address the health needs of adolescents and increase their participation in health services^45^.

### Limitation

Self-evaluation, when used isolated, does not provide insight into other perspectives or indicators for evaluating teaching programs. However, the high participation rate of nursing schools and the results presented here are important indicators of nursing education in some Latin America countries.

### Implications for knowledge advancement

The results indicated limitations in terms of teaching capacity to train nurses, thus indicating the need to invest in the continuous education of nurses and faculty regarding pedagogical updating and adolescent health.

The knowledge gaps identfied can be used to develop continuing and permanent training courses, according to the reality of each country, and encourage the development of scientific production in adolescent health among nurses.

Finally, the results reinforce the importance of advancing school health and implementing effective measures for interaction between health services and schools. Nurses, in their different fields of action, must advocate for adolescent health, expanding their capabilities and leading actions.

## Conclusion

Analyzing the structure and content of the teaching programs in the three countries allowed us to draw parallels and make inferences for other Latin American countries.

The lack of adequate educational/pedagogical preparation and knowledge on adolescent health limits the effective implementation of the teaching program by faculty.

The advancement of strategies to understand and establish effective communication with this population is necessary in nursing education. Nurses and nursing students need to understand health/epidemiological data, laws, and policies to guide their decision making.

The revision of the curricula and inclusion of current and relevant topics of adolescent health and development should incorporate adolescent-centered topics such as: behavior (decision and attitude), gender identity and sexual orientation, bullying and cyberbullying, use of digital technologies, partner violence, adolescent parenthood, puberty delay.

Finally, health enhancement in this population from the nursing perspective can be achieved by strategies that include the implementation of school health and effective measures of interaction between health services and schools.

## References

[1] World Health Organization (2022). Working for a brighter, healthier future: how WHO improves health and promotes well-being for the world’s adolescents.

[2] Organización Mundial de la Salud (2015). Competencias básicas en materia de salud y desarrollo de los adolescentes para los proveedores de atención primaria: incluido un instrumento para evaluar el componente de salud y desarrollo de los adolescentes en la formación previa al servicio de los proveedores de atención sanitaria.

[3] Manzanero JRL. (2021). Youth in Latin America and the Caribbean in perspective: overview of the situation, challenges and promising interventions. Cien Saude Colet.

[4] Santana KC, Silva EKP, Rodriguez RB, Bezerra VM, Souzas R, Medeiros DS. (2021). Health service utilization by Quilombola and non-Quilombola adolescents living in a rural area in the semi-arid region of the state of Bahia, Brazil. Cien Saude Colet.

[5] United Nations Population Fund, World Health Organization, International Confederation of Midwives (2021). The State of the World’s Midwifery 2021.

[6] Pan American Health Organization (2018). Plan of Action for Women’s, Children’s, and Adolescents’ Health 2018-2030.

[7] (2021). World Health Organization. Global strategic directions for nursing and midwifery 2021-2025.

[8] Cassiani SHDB, Wilson LL, Mikael SSE, Peña LM, Grajales RAZ, McCreary LL (2017). The situation of nursing education in Latin America and the Caribbean towards universal health. Rev. Latino-Am. Enfermagem.

[9] Chen T, Peng L, Yin X, Rong J, Yang J, Cong G. (2020). Analysis of User Satisfaction with Online Education Platforms in China during the COVID-19 Pandemic. Healthcare.

[10] Scott J, Johnson R, Ibemere S. (2021). Addressing health inequities re-illuminated by the COVID-19 pandemic: How can nursing respond. Nurs Forum.

[11] Carmo TRG, Santos RL, Magalhães BC, Silva RA, Dantas MB, Silva VM. (2021). Competencies in health promotion by nurses for adolescents. Rev Bras Enferm.

[12] Organización Panamericana de la Salud (2021). Observatorio Regional de Recursos Humanos de Salud.

[13] World Health Organization (2020). State of the World’s Nursing Report.

[14] Muraraneza C, Mtshali GN. (2021). Planning reform to competency based curricula in undergraduate nursing and midwifery education: A qualitative study. Nurse Educ Today.

[15] Bogren M, Doraiswamy S, Erlandsson K, Akhter H, Akter D, Begum M (2018). Development of a context specific accreditation assessment tool for affirming quality midwifery education in Bangladesh. Midwifery.

[16] Al-Alawi R, Alexander GL (2020). Systematic review of program evaluation in baccalaureate nursing programs. J Prof Nurs.

[17] McCarthy B, Trace A, O’Donovan M, Brady-Nevin C, Murphy M, O’Shea M (2018). Nursing and midwifery students’ stress and coping during their undergraduate education programmes: An integrative review. Nurse Educ Today.

[18] Dionne Merlin M, Lavoie S, Gallagher F. (2020). Elements of group dynamics that influence learning in small groups in undergraduate students: A scoping review. Nurse Educ Today.

[19] Gill Meeley N. (2021). Undergraduate student nurses’ experiences of their community placements. Nurse Educ Today.

[20] Fogg N, Wilson C, Trinka M, Campbell R, Thomson A, Merritt L (2020). Transitioning from direct care to virtual clinical experiences during the COVID-19 pandemic. J Prof Nurs.

[21] Tawalbeh LI. (2020). Effect of simulation modules on Jordanian nursing student knowledge and confidence in performing critical care skills: A randomized controlled trial. Int J Africa Nurs Sci.

[22] Booth RG, Strudwick G, McBride S, O’Connor S, Solano López AL. (2021). How the nursing profession should adapt for a digital future. BMJ.

[23] World Health Organization (2020). Digital education for building health workforce capacity.

[24] Ramos-Morcillo AJ, Leal-Costa C, Moral-García JE, Ruzafa-Martínez M. (2020). Experiences of Nursing Students during the Abrupt Change from Face-to-Face to e-Learning Education during the First Month of Confinement Due to COVID-19 in Spain. Int J Environ Res Public Health.

[25] Peixoto AMCL, Melo TQ, Ferraz LAA, Santos CFBF, Godoy F, Valença PAM (2021). Demand for health services or professionals among adolescents: a multilevel study. Cien Saude Colet.

[26] Neves JVVS, Carvalho LA, Carvalho MA, Silva ETC, Alves MLTS, Silveira MF (2021). Alcohol use, family conflicts and parental supervision among high school students. Cien Saude Colet.

[27] Bottino SMB, Bottino CMC, Regina CG, Correia AVL, Ribeiro WS. (2015). Cyberbullying and adolescent mental health: systematic review. Cad Saude Publica.

[28] John A, Glendenning AC, Marchant A, Montgomery P, Stewart A, Wood S (2018). Self-Harm, Suicidal Behaviours, and Cyberbullying in Children and Young People: Systematic Review. J Med Internet Res.

[29] Hamm MP, Newton AS, Chisholm A, Shulhan J, Milne A, Sundar P (2015). Prevalence and Effect of Cyberbullying on Children and Young People. JAMA Pediatr.

[30] Dienlin T, Johannes N. (2020). The impact of digital technology use on adolescent well-being. Dialogues Clin Neurosci.

[31] Nunes PPB, Abdon APV, Brito CB, Silva FVM, Santos ICA, Martins DQ (2021). Factors related to smartphone addiction in adolescents from a region in Northeastern Brazil. Cien Saude Colet.

[32] Carvalhaes RS, Cárdenas CMM (2021). “Dating is pure suffering”: violence within affective-sexual relationships between adolescents in a school in the Costa Verde, Rio de Janeiro, Brazil. Cien Saude Colet.

[33] Ferrari W, Nascimento MAF, Nogueira C, Rodrigues L. (2021). Violence in the affective-sexual trajectories of young gay men: “new” settings and “old” challenges. Cien Saude Colet.

[34] Vieira MF, Deslandes SF, Gomes SCS (2021). Know-how and techniques of Family Health Strategy managers and professionals in the prevention of violence against adolescents. Cien Saude Colet.

[35] Sardinha L, Maheu-Giroux M, Stöckl H, Meyer SR, García-Moreno C. (2022). Global, regional, and national prevalence estimates of physical or sexual, or both, intimate partner violence against women in 2018. Lancet.

[36] Bamishigbin ON, Dunkel Schetter C, Stanton AL. (2019). The antecedents and consequences of adolescent fatherhood: a systematic review. Soc Sci Med.

[37] Recto P, Lesser J. (2021). Adolescent Fathers’ Perceptions and Experiences of Fatherhood: A Qualitative Exploration with Hispanic Adolescent Fathers. J Pediatr Nurs.

[38] Sultan C, Gaspari L, Maimoun L, Kalfa N, Paris F. (2018). Disorders of puberty. Best Pract Res Clin Obstet Gynaecol.

[39] Silva JCP, Cardoso RR, Cardoso ÂMR, Gonçalves RS. (2021). Sexual diversity: a perspective on the impact of stigma and discrimination on adolescence. Cien Saude Colet.

[40] Rider GN, McMorris BJ, Gower AL, Coleman E, Eisenberg ME (2018). Health and Care Utilization of Transgender and Gender Nonconforming Youth: A Population-Based Study. Pediatrics.

[41] Ozturk FO, Ayaz-Alkaya S. (2020). Health Literacy and Health Promotion Behaviors of Adolescents in Turkey. J Pediatr Nurs.

[42] Caldwell EP, Melton K. (2020). Health Literacy of Adolescents. J Pediatr Nurs.

[43] Hilli Y, Pedersen G. (2021). School nurses’ engagement and care ethics in promoting adolescent health. Nurs Ethics.

[44] Best NC, Nichols AO, Waller AE, Zomorodi M, Pierre-Louis B, Oppewal S (2021). Impact of School Nurse Ratios and Health Services on Selected Student Health and Education Outcomes: North Carolina, 2011-2016. J Sch Health.

[45] Daley AM, Polifroni EC, Sadler LS (2019). The Essential Elements of Adolescent-friendly Care in School-based Health Centers: A Mixed Methods Study of the Perspectives of Nurse Practitioners and Adolescents. J Pediatr Nurs.

